# Mind Games: When Neurosyphilis and Borderline Personality Disorder Collide – A Diagnostic Puzzle

**DOI:** 10.1192/j.eurpsy.2025.533

**Published:** 2025-08-26

**Authors:** A. G. Buciuc, A. D. Gurfinchel Zevallos, M. Hadjikyriakou

**Affiliations:** 1 Psychiatry, University of Miami/ Jackson Health System; 2 Psychiatry and Behavioral Sciences, Bruce W. Carter Department of Veterans Affairs Medical Center, Miami, United States

## Abstract

**Introduction:**

Historically, syphilis has been known as “the Great Imitator” due to its heterogeneous clinical manifestations. Though its incidence decreased with the widespread use of penicillin, recent data suggest a resurgence, particularly among those experiencing delays in treatment. This resurgence creates diagnostic challenges, especially when patients have coexisting psychiatric conditions like Borderline Personality Disorder (BPD).

**Objectives:**

This report explores the psychiatric and cognitive manifestations of neurosyphilis in patients with preexisting personality disorders. The primary objective is to highlight how neurosyphilis complicates psychiatric diagnosis and care and to emphasize the need for early detection and intervention.

**Methods:**

A thorough literature review was conducted using PubMed database covering studies published from 2000 to 2023. Keywords included “neurosyphilis,” “borderline personality disorder,” “psychiatric symptoms,” “syphilis resurgence,” and “cognitive impairment.” Additionally, the case of a 35-year-old female with BPD who developed neurosyphilis is presented, demonstrating the complexities in distinguishing between overlapping psychiatric symptoms. The case also emphasizes the importance of comprehensive care.

**Results:**

The psychiatric symptoms of neurosyphilis, such as impulsivity, mood instability, and cognitive dysfunction, significantly overlap with those of BPD, complicating diagnosis and treatment. Literature indicates that neurosyphilis occurs in 0.5% to 2% of untreated syphilis cases. Common psychiatric manifestations of neurosyphilis—such as irritability, cognitive decline, and affective dysregulation—are often misattributed to underlying psychiatric disorders, leading to delays in proper treatment. In the case of the 35-year-old patient, her longstanding BPD symptoms, including emotional instability and impulsivity, worsened with the progression of neurosyphilis. Cognitive testing revealed mild impairment, which was consistent with the cognitive decline seen in neurosyphilis, further complicating the clinical picture.

**Conclusions:**

This case underscores the critical need for timely syphilis screening, particularly for individuals with a history of untreated or inadequately treated infections. Early diagnosis and treatment of neurosyphilis can significantly improve cognitive and psychiatric outcomes while promoting overall wellness. Routine sexually transmitted disease screenings, especially in psychiatric populations, can prevent severe neuropsychiatric complications and support holistic well-being. Given the global resurgence of syphilis, a proactive approach to sexual health is essential in fostering both mental and physical health.

**Disclosure of Interest:**

None Declared

